# Origami nanogap electrodes for reversible nanoparticle trapping†

**DOI:** 10.1039/d4nr00190g

**Published:** 2024-03-22

**Authors:** Itir Bakis Dogru-Yuksel, Allard P. Mosk, Sanli Faez

**Affiliations:** a Nanophotonics, Debye Institute for Nanomaterials Science, Utrecht University 3584 CC Utrecht The Netherlands i.b.dogruyuksel@uu.nl s.faez@uu.nl

## Abstract

We present a facile desktop fabrication method for origami-based nanogap indium tin oxide (ITO) electrokinetic particle traps, providing a simplified approach compared to traditional lithographic techniques and effective trapping of nanoparticles. Our approach involves bending ITO thin films on optically transparent polyethylene terephthalate (PET), creating an array of parallel nanogaps. By strategically introducing weak points through cut-sharp edges, we successfully controlled the spread of nanocracks. A single crack spanning the constriction width and splitting the conductive layers forms a nanogap that can effectively trap small nanoparticles after applying an alternating electric potential across the nanogap. We analyze the conditions for reversible trapping and optimal performance of the nanogap ITO electrodes with optical microscopy and electrokinetic impedance spectroscopy. Our findings highlight the potential of this facile fabrication method for the use of ITO at active electro-actuated traps in microfluidic systems.

## Abstract 2

Drawing inspiration from the time-honored origami technique, we crafted electrodes from a conductive plastic sheet in a single step, enabling the reversible trapping of nanoparticles for analysis within the image plane.

## Graphical abstract

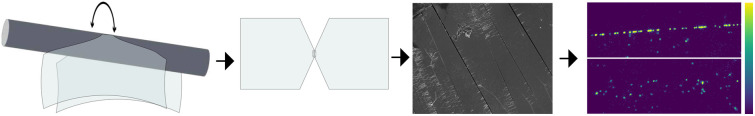

Potentiodynamic manipulation and steering of nanoparticles in microfluidic systems is one of the key design elements for lab-on-chip applications. Using alternating electrical potential for nanoparticle trapping is advantageous for the higher electrochemical stability of the electrodes. The main mechanisms that have been used to achieve stable and reversible trapping of nanoparticles are dielectrophoresis (DEP) and alternating current electroosmotic (ACEO) flow,^1^ which may coexist in some geometries. Dielectrophoresic forces arise from the interaction between the induced dipole moment of dielectric particles and the local gradient of the electric field in the surrounding environment.^2^ This interaction results in a net force that can be used to attract and trap particles within a non-uniform electric field near an electrode.^3,4^ For nanoscale localization and trapping of nanoparticles with DEP, *e.g.* to overcome the Brownian forces, the gradient must be significant on the scale of nanoparticles and that of the desired trapping area.^5^

Compared to DEP, ACEO trapping demonstrates increased complexity, as it depends on 3D flow generated on top of the electrodes, particularly around edges or at the inter-junction between conducting electrodes and charged dielectric surfaces. This phenomenon often creates some net vorticity in the fluid flow that can draw the particles towards the junction or can be used for fluid mixing and creating an electroosmotic pump.^6,7^ Both DEP and ACEO phenomena have been used for creating potentiodynamic nanoparticle traps and are widely used in lab-on-chip systems for the precise measurement,^8,9^ separation,^10–12^ sorting,^13,14^ and manipulation^15–17^ of suspended particles in liquid media. Because of electrical control and the possibility of system integration, potentiodynamic manipulation techniques also enable accurate single-cell manipulation, contributing to advancements in biomedical diagnostics^18–20^ and therapy^21,22^ and featuring promising prospects for the development of a point-of-care tool in the future.^23^ For both mechanisms, the key to realization of a sufficiently strong trap for small nanoparticles is to create a large gradient, hence a small radius of curvature at the electrode and small electrode separations are crucial^24^ and the effective force is inversely proportional to the gap size for micro-structured planar electrodes.^25^ Creating ACEO and DEP traps that are compatible with optical microscopy can further constrain the choice of materials and require a meticulous design of the nanoelectrodes. Crafting robust electrodes with nanoscale edges demands precise engineering and is often done with nanolithographic techniques to achieve trapping of, for example, sub-30 nm (bio)particles. Barik *et al.* employed atomic layer lithography to generate nanoscale gaps.^26^ Han *et al.* utilized electron beam lithography for fabricating an electrode array.^27^ Yu *et al.* employed photolithography to construct vertical nanogap architectures, showcasing precise nanoparticle capture and spatiotemporal manipulation.^28^ While nanolithography provides a systemic path to the optimization of such potentiodynamic traps, it is not accessible to all labs and requires specialised personnel and equipment. In contrast, our method's simplicity relative to lithography presents an advantage, albeit with a tradeoff: the precise landing location of particles in the plane is not predetermined. However, our simple method brings particles to the focus region from the bulk, and localization of particles within the image plane must be done with (live) image processing. The capability to trap particles extends observation time, a key factor in studying dynamic processes.

In this article, we demonstrate an innovative method for generating nanoparticle-trapping nanogap electrodes that remarkably requires only regular office equipment. Our traps use the origami technique, involving the straightforward manipulation of thin conductive layers through bending. Our nanogap electrode production protocol stands out for its simplicity, speed, and reproducibility and can enable a wide community of researchers to use the advantages of electro-actuated trapping for their lab-on-chip applications.

This fabrication method is inspired by previous work on origami-fabrication of quasi-ordered nanocracks within protein layers, proposed as a mechanism for building distributed feedback bio-compatible lasers.^29^ We apply this fabrication method to a thin layer of indium tin oxide (ITO) coated on PET (polyethylene terephthalate). This substrate is widely available and affordable because of its commercial application for liquid crystal displays and devices. By implementing controlled crack propagation, a phenomenon extensively investigated before,^30–37^ we demonstrate reversible trapping and particle alignment dynamics. The transparency of the substrate holds the potential to facilitate detailed examinations of biological structures, down to the level of individual cells or sub-cellular structures.

## 1. Results

### 1.1. Nanogap ITO electrodes

**Fig. 1:**
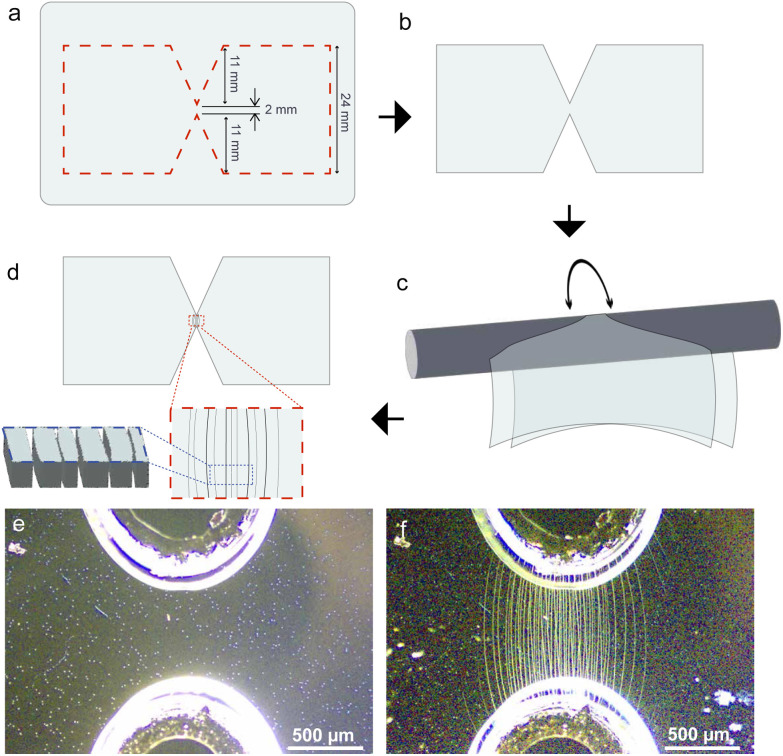
Fabrication of nanogap origami electrodes using ITO on PET: (a) the laser-cut design, with dashed red lines indicating the cut sections. (b) The cut layer after the laser-cutting process. (c) The process of fabricating origami nanocracks by bending the material around a metal rod. (d) The resulting nanocracks formed in the mid-region, shown in both the top view (highlighted by the red dashed lines) and a magnified cross-sectional perspective (indicated by the blue dashed lines). An optical microscopy image of the nanocrack fabrication region (e) before and (f) after bending.

We used a combination of laser-cutting and controlled bending to fabricate our origami nanogap electrodes. The substrate used for this study is a 90–150 Å ITO layer deposited on 200 μm PET with a resistance range of 
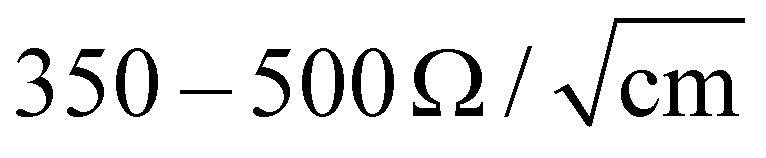
. The substrate's flexibility is essential for the fabrication of origami-based electrodes, as it can be easily bent and manipulated to achieve the desired structure. We first cut a butterfly-shaped piece from the ITO-PET film. On purpose, we introduced weak points in the mid-region, where small triangles point towards the center with a 2 mm spacing as illustrated in Fig. 1a and as depicted in Fig. 1b. This design reduces the likelihood of crack propagation further from the thin bridge. The resulting cracks align parallel to each other, which increases the probability of their completeness without overlapping with adjacent gaps. While laser cutting is handy for reproducibility, we have successfully replicated these experiments using scissor-cut samples. For the purpose of reproducibility, we avoid using scissor cutting, which might create additional cracks at the cut edges. Remarkably, the presence of a single complete crack that spans the whole width of the narrow constriction and effectively divides the conductive layer into at least two planar sections is sufficient to form the nanogap trap for nanoparticles. To apply uniform stress, we used a metal rod with a diameter of 60 mm to gently bend the PET film and roll it (see Fig. 1c). After this step, nanocracks are visible under an optical microscope. They are formed in parallel to each other as illustrated in Fig. 1d. Fig. 1e and f present the state before and after bending, respectively.

The gap size can be estimated by a simple mode that considers the difference in the circumferences of the tangent circles on top and bottom of the rounded film. The separation between adjacent cracks is relatively constant in the investigation area at *w*_r_ ≈ 15.5 ± 4.4 μm (Fig. S1a†). The nanogap size is given by *d*_g_ = *t*_f_*w*_r_/*R*, where *R* is the bending radius and *t*_f_ is the PET-substrate film thickness. Using *R* = 30 mm and *t*_f_ = 200 μm, we estimated an average gap opening of *d*_g_ = 100 ± 30 nm, which is smaller than the gap size, that is 258 ± 95 nm, measured with electron microscopy (Fig. S1b†). This measurement aligns with the estimated value, which falls within the observed range. Empirically, we observed that the exact gap size, within the range of our samples, is not correlated with trapping efficiency. This indicated that at the low AC frequencies that we used, the main potential drop is across the electric double layer. Therefore, controlling the exact size is of minor importance. Our simple model considers only geometric effects and does not include the possible influence of possible shear tension between the ITO film and the PET substrate or the irreversible deformation of the PET substrate after bending. This simple model, however, shows how to control the gap size of the origami nanocrack electrodes by controlled bending of the substrate.

### 1.2. Reversible potentiodynamic trapping

**Fig. 2:**
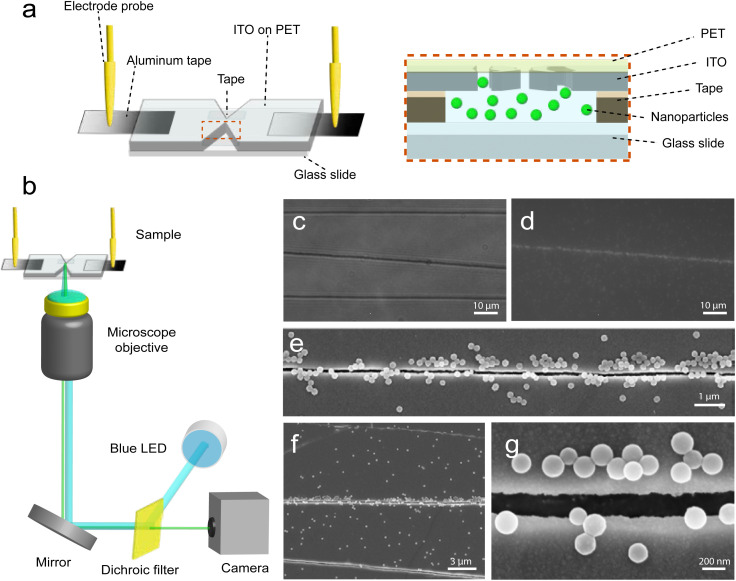
(a) Schematic representation of an inverted nanocrack electrode configuration, where nanoparticles in water are confined between the electrode and a glass slide, and an electric field is generated by aluminum tape and gold electrodes. (b) Schematic of a custom-built fluorescence microscopy setup and a wavefunction generator connected to the electrode probes. (c) Visualizing origami nanocracks under white light and (d) detecting particles trapped within a nanocrack using fluorescence. (e), (f) and (g) SEM images depicting particles concentrated at the nanocrack edges, captured at various magnifications.

The origami nanogap electrode was affixed to a glass slide using double-sided tape. Electric contacts were made with aluminum tape to facilitate the attachment of probing electrode rods from both sides (Fig. 2a). We emphasize that our samples, because of the transparent substrate, are compatible with any upright or inverted microscope. We used 200 nm fluorescent polystyrene (PS) nanoparticles in water to demonstrate reversible trapping around the gaps. To visualize the particles, we used a lab wavefunction generator to apply the trapping potential and a custom-built fluorescence microscopy setup, with a blue LED for illumination, to observe their response (Fig. 2b). Effective trapping occurs when the electrokinetic forces surpass counteracting forces like thermal Brownian motion or convection.^38^ When cracks are shorter than the cross section, the negligible voltage drop due to incomplete electric conduction has no observable influence on the particle motion. Incomplete cracks remain conductive with negligible potential drop, and trapping predominantly occurs along a subset of cracks. The scanning electron microscopy (SEM) images in Fig. S2† provide additional images confirming the completeness of the trapping origami nanocrack. This effect is highlighted in the bright-field image of three cracks in Fig. 2c. Moreover, the fluorescence microscopy image in Fig. 2d from the same region as Fig. 2c clearly shows that incomplete cracks do not achieve the desired effect. The SEM images in Fig. 2e, f and g demonstrate the adhesion of particles on the edges of the crack that cover the entire cross-section of the constriction under specific potential parameters.

**Fig. 3:**
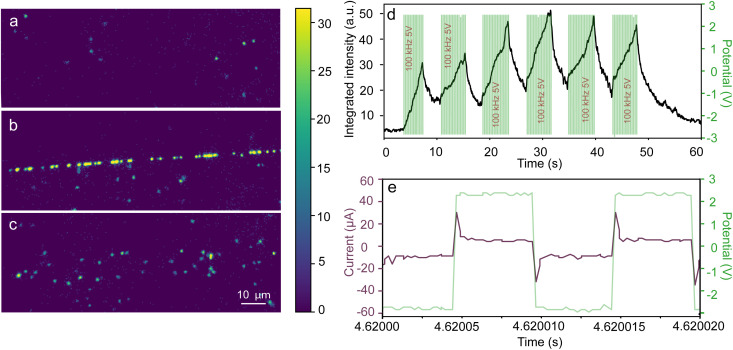
Dynamic stages of particles in water within nanocracks: (a) before, (b) during, and (c) after the application of an electric field. (d) Integrated intensity change over time (black) is correlated with the applied potential (green). (e) A close-up view of the applied potential (green) with 100-microsecond resolution, alongside simultaneous recording of the electric current passing through the nanocrack samples (purple).

Before applying the electric field, we observe the particles exhibiting Brownian motion, as depicted in Fig. 3a. When the electric field is first applied (a 5 V peak-to-peak rectangular waveform at 100 kHz), the particles are drawn to and remain localized in the nanocrack region, as illustrated in Fig. 3b. However, upon deactivation of the field, the particles are promptly released, as shown in Fig. 3c (also see ESI Video 1†). The rapid response of particles to an applied electric field is assessed by measuring the integrated intensity change along a nanocrack. The integrated intensity undergoes a rapid increase upon applying the electric field, followed by a relatively slower decline when the potential is set back to zero, as depicted in Fig. 3d. The green data represent the applied potential, demonstrating that the AC electric potential is intermittently applied for a 5-second time interval, repeated six times with 3-second intervals of zero potential. The zoomed-in view in Fig. 3e, focusing on an applied potential of 5 V at 100 kHz with a temporal resolution of 100 microseconds, enables a detailed analysis of the electric field dynamics while the purple data show the current measured from the nanocracks.

**Fig. 4:**
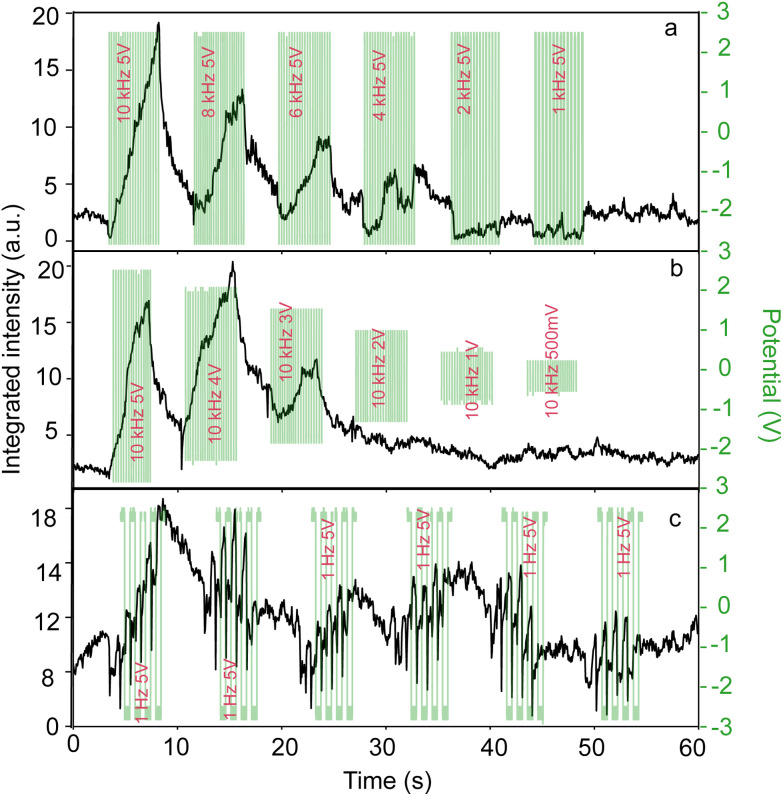
The integrated intensity change over time (a) as the frequency is systematically decreased, (b) when the applied voltage is systematically decreased and (c) at 5 V with 1 Hz application.

Under a 5 V peak-to-peak amplitude of the applied potential, we gradually reduced the frequency from 100 kHz to 1 Hz (see also ESI Video 2†). It is worth mentioning that when two complete cracks are present, we observed simultaneous trapping occurring within these parallel cracks as shown in Fig. S3.† The nuanced dynamics of reversible trapping at 10 000 Hz is further elucidated in Fig. S4,† resembling Fig. 3, but with a diminished integrated intensity. Nanoparticle trapping persisted at all frequencies above 2 kHz. We observed no trapping at frequencies lower than 2 kHz (Fig. 4a). The specifics of particle behavior spanning from 100 kHz to 1 Hz (100 kHz, 10 kHz, 1 kHz, 100 Hz, 10 Hz, and 1 Hz) are presented in Fig. S5.†

Conversely, when holding the frequency at 10 kHz and varying the amplitude (5, 4, 3, 2, 1 V, and 500 mV), a distinct pattern emerged (Fig. 4b). Trapping effects were negligible at or below 2 V, while reversible trapping was observed at higher voltages (see ESI Video 3†).

Intriguingly, under the application of 5 V at 1 Hz (cycled six times over 5 seconds with 3-second intervals), particles exhibited a unique response. They displayed an oscillatory motion, shuttling between the sides of the nanocrack line in sync with the applied potential. This dynamic movement is clearly depicted in Fig. 4c, aligning with the peak and dip points corresponding to the application of the electric field, which is a clear indication of the bulk-flow induced by applying the electric potential. However, the cycle-averaged forces at these low frequencies are not enough to concentrate or eventually trap the nanoparticles (see also ESI Video 4†).

The observed electrokinetic trapping cannot be explained by DEP as we expect positive (repulsive from the electrode) forces on polystyrene particles at low frequencies. The DEP forces are expected to change sign at frequencies above 100 kHz for the conditions of our sample.^1^ However, we can observe trapping at much lower frequencies, even down to 2 kHz. However, ACEO trapping has been observed at lower frequencies, which we anticipate is the underlying mechanism for trapping the nanoparticles in our system. To explore these trapping conditions and the stability of the sample empirically, we conducted a parametric study of the applied potential and frequencies using cyclic voltammetry and electrochemical impedance spectroscopy (EIS) of the same nanogap electrodes in contact with the nanoparticle sample or other electrolyte solutions.

### 1.3. Impedance spectroscopy of origami nanocrack electrodes

In order to characterize and understand the ideal trapping conditions and check the reproducibility of each fabricated electrode, we used electrochemical impedance spectroscopy (EIS). In this method, the complex-valued conductivity of the sample is measured as a function of frequency and amplitude for a sinusoidal AC potential, revealing the various conduction regimes in the electrolyte. To understand the influence of the electric-double-layer formation at the electrodes, we also repeated our characterization for a series of ionic strengths in solutions of increasing potassium chloride (KCl) concentration.

**Fig. 5:**
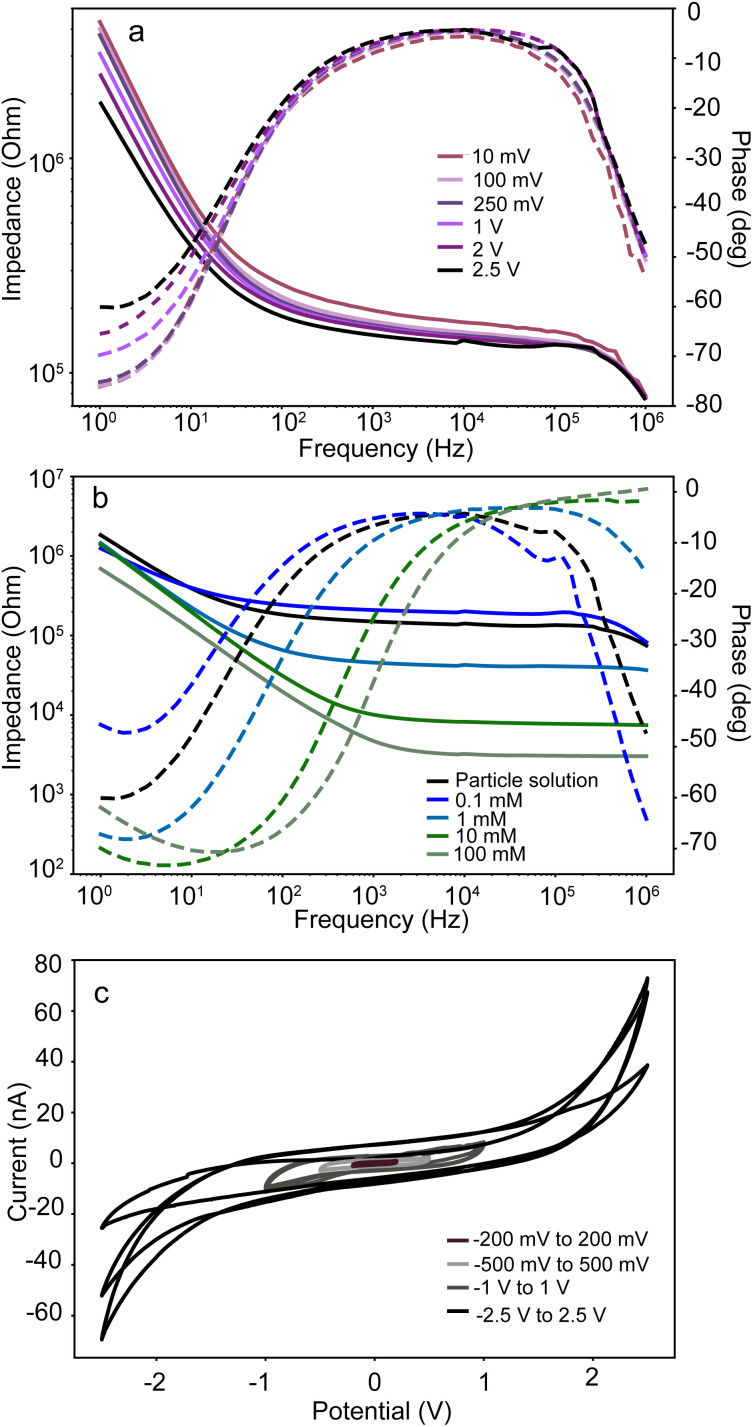
Bode plots of the impedance of the ITO electrodes for sinusoidal alternating potential (a) in contact with the nanoparticle solution for varying wave amplitude and (b) in contact with a KCl solution of different concentrations. (c) Cyclic voltammetry of ITO electrodes in contact with the nanoparticle solution for different ranges of the applied potential. For each range, 3 cycles have been measured. Only the highest range displays some alterations in the measured current for the extremes of the potential that can be attributed to the electrochemical change in the surface properties such as roughness.

Fig. 5(a) depicts the measured impedance spectrum of the ITO electrodes in contact with the nanoparticle suspension for various applied potentials. We can observe little difference in the impedance response, especially at frequencies above 100 Hz, which testifies to the electrochemical stability of the ITO substrate.

In Fig. 5(b), we present the EIS measurements for the ITO electrodes for various concentrations of KCl dissolved in water at 2.5 V. We can observe that conductivity increases (low-frequency impedance decreases) with higher salt concentrations. Furthermore, the response phase at the highest frequencies deviates from zero for the lowest measured salt concentration of 0.1 mM, for which the charging time of the electric double layer becomes comparable to the period of the waveform. The response of the nanoparticle solution is closest to that of the 0.1 mM salt concentration. We therefore conclude that the residual ionic strength of the nanoparticle solution used for our measurements is in that range. The cyclic voltammetry diagram of origami electrodes with particle solution depicted in Fig. 5(c) also confirms the electrochemical stability for the potential range of ±2.5 V. Water splitting reactions speed up outside this range, resulting in higher faradaic currents, but this effect seems to be negligible for AC potentials at frequencies larger than 100 Hz that are suitable for trapping at the nanogap, which is the main focus of this article. The absence of faradaic reactions is advantageous for the main function of nanogap trapping as it enhances the chemical stability of the ITO electrode. The stability conditions, however, are only tested at neutral pH. The use of acidic or basic solutions might further limit the usability of this device.

## 2. Conclusions

In this study, we introduce a novel approach for nanogap electrode fabrication, utilizing origami principles to create controlled nanocracks in transparent and conductive films. This method deviates from conventional lithographic techniques, offering a point-of-use one-step process for the production of parallel-arranged nanogap electrode patterns. The stress-induced merging of strategically introduced weak points results in reversible nanoparticle confinement, as evidenced by the observed dynamics under varying parameters such as the amplitude and frequency of the applied waveform. The trapping behavior persists at AC frequencies as low as 2 kHz, consistent with the hypothesis of AC electroosmosis as the main underlying mechanism. Based on EIS measurements, we demonstrate the stability of ITO electrodes for the relevant potentials and frequencies and rule out the influence of faradaic reactions at neutral pH, which could otherwise result in chemical degradation.

These nanogap electrodes can be used in various applications, including integration into lab-on-a-chip systems for controlled analysis of biological particles, microfluidic devices for efficient particle transport and sorting, and biotechnology applications for the manipulation of cells and biomolecules.

## 3. Materials and methods

### 3.1. Nanogap ITO electrode fabrication

90–150 Å ITO coated PET films with a thickness of 200 μm (OCF2520, Thorlabs) were precision-cut using a GCC LaserPro X380 laser engraver/cutter according to a vectorial program defining the electrode design. The resulting electrodes measured 24 mm in height and 50 mm in length, featuring weak points in the mid-region with small triangles pointing towards the center, spaced at 2 mm intervals in a ribbon shape. Nanocracks were induced in the ITO-coated PET film by bending it around metal rods with a constant 30 mm radius of curvature. The longitudinal strain applied to the film surface during smooth rolling resulted in uniformly spaced nanocracks with an average of 15.5 μm intervals in the mid-region. The electrodes were inverted and affixed to a 24 × 50 mm no. 1.5 glass slide using small square pieces of double-sided tape. The ITO conductivity was enhanced with aluminum tape, facilitating the attachment of electrode probes from both sides. This assembly effectively confined the particle solution between the glass slide and the electrode design, minimizing the impact of water evaporation and drift.

### 3.2. Particle solution preparation

Aqueous suspensions of 0.20 μm Fluoresbrite® YG carboxylate microspheres (2.5% w/v) with a maximum excitation of 441 nm and a maximum emission of 486 nm were diluted 10 000 times with Milli-Q water. For trapping experiments, 60 μl of the diluted solution was used.

### 3.3. Trapping experiments

For electrokinetic trapping measurements, we used a function generator and a home-built fluorescence microscopy setup. A blue LED (Thorlabs, M395L4) was employed to excite the particles, with the excitation light reflected by a dichroic filter directed to the microscope objective (ZEISS 40×/0.65) *via* a mirror. The emitted light followed the reverse path, passing through a long-pass dichroic filter (500 nm) and reaching the camera (Hamamatsu Digital Camera, C11440). This configuration effectively filtered the source light from the image, enabling particle visualization. A waveform generator (KEYSIGHT InfiniiVision DSOX2024A) synchronized with the camera monitored the applied voltage and circuit, facilitating simultaneous observation of the impact of the applied potential on particle behavior.

### 3.4. Impedance spectroscopy

A BioLogic SP300 potentiostat was employed for impedance and cyclic voltammetry measurements of the origami nanocrack electrodes.

### 3.5. Characterization

Nanocrack morphology and trapped PS particles were analyzed using a ZEISS EVO 15 SEM operated at 5 kV.

## Author contributions

Itir Bakis Dogru-Yuksel: visualization, investigation, conceptualization, methodology, and writing – original draft preparation. Allard P. Mosk: validation, writing – reviewing and editing, and supervision. Sanli Faez: conceptualization, methodology, writing – original draft preparation, writing – reviewing and editing, and supervision.

## Conflicts of interest

There are no conflicts to declare.

## Supplementary Material

NR-016-D4NR00190G-s001

NR-016-D4NR00190G-s002

NR-016-D4NR00190G-s003

NR-016-D4NR00190G-s004

NR-016-D4NR00190G-s005

## References

[cit1] Wang J., Wei M.-T., Cohen J. A., Ou-Yang H. D. (2013). Electrophoresis.

[cit2] Lu Y.-W., Sun C., Kao Y.-C., Hung C.-L., Juang J.-Y. (2020). Nanomaterials.

[cit3] Sun H., Ren Y., Hou L., Tao Y., Liu W., Jiang T., Jiang H. (2019). Anal. Chem..

[cit4] Zaman M. A., Padhy P., Ren W., Wu M., Hesselink L. (2021). J. Appl. Phys..

[cit5] Pandey P., Panday N., Chang S., Pang P., Garcia J., Wang X., Fu Q., He J. (2018). ChemElectroChem.

[cit6] Squires T. M. (2009). Lab Chip.

[cit7] Zhang Z., de Graaf J., Faez S. (2020). Soft Matter.

[cit8] Pakhira W., Kumar R., Ibrahimi K. M., Bhattacharjee R. (2022). J. Braz. Soc. Mech. Sci. Eng..

[cit9] Liu Z., Frijns A. J., Speetjens M. F., van Steenhoven A. A. (2015). Microfluid. Nanofluid..

[cit10] Hughes M. P. (2002). Electrophoresis.

[cit11] Liu D., Song B., Chen L., Sun L. (2011). Mech. Eng. J..

[cit12] Zhou H., White L. R., Tilton R. D. (2005). J. Colloid Interface Sci..

[cit13] Boettcher M., Jaeger M. S., Riegger L., Ducrée J., Zengerle R., Duschl C. (2006). Biophys. Rev. Lett..

[cit14] Islam N., Wu J. (2006). J. Phys.: Conf. Ser..

[cit15] Miled M. A., Sawan M. (2012). IEEE Trans. Biomed. Circuits Sys..

[cit16] Dalili A., Taatizadeh E., Tahmooressi H., Tasnim N., Rellstab-Sánchez P. I., Shaunessy M., Najjaran H., Hoorfar M. (2020). Sci. Rep..

[cit17] Hilber W., Weiss B., Mikolasek M., Holly R., Hingerl K., Jakoby B. (2008). J. Micromech. Microeng..

[cit18] Lambert E., Manczak R., Barthout E., Saada S., Porcù E., Maule F., Bessette B., Viola G., Persano L., Dalmay C. (2021). et al.. Biosensors.

[cit19] Hochstetter A. (2020). Micromachines.

[cit20] Chuang C.-H., Wu T.-F., Chen C.-H., Chang K.-C., Ju J.-W., Huang Y.-W., Van Nhan V. (2015). Lab Chip.

[cit21] Çağlayan Z., Demircan Yalçın Y., Külah H. (2020). Micromachines.

[cit22] Ivanoff C. S., Swami N. S., Hottel T. L., Garcia-Godoy F. (2013). Electrophoresis.

[cit23] Demircan Y., Özgür E., Külah H. (2013). Electrophoresis.

[cit24] Chiou P. Y., Ohta A. T., Wu M. C. (2005). Nature.

[cit25] Cheng P., Barrett M. J., Oliver P. M., Cetin D., Vezenov D. (2011). Lab Chip.

[cit26] Barik A., Chen X., Oh S.-H. (2016). Nano Lett..

[cit27] Han J., Niroui F., Lang J. H., Bulovic V. (2022). Nano Lett..

[cit28] Yu E.-S., Lee H., Lee S.-M., Kim J., Kim T., Lee J., Kim C., Seo M., Kim J. H., Byun Y. T. (2020). et al.. Nat. Commun..

[cit29] Dogru-Yuksel I. B., Jeong C., Park B., Han M., Lee J. S., Kim T.-i., Nizamoglu S. (2021). Adv. Funct. Mater..

[cit30] Park B., Kim J., Kang D., Jeong C., Kim K. S., Kim J. U., Yoo P. J., Kim T.-i. (2016). Adv. Mater..

[cit31] Jung Y. H., Park B., Kim J. U., Kim T.-i. (2019). Adv. Mater..

[cit32] Park B., Kim J. U., Kim J., Tahk D., Jeong C., Ok J., Shin J. H., Kang D., Kim T.-i. (2019). Adv. Funct. Mater..

[cit33] Suh Y. D., Yeo J., Lee H., Hong S., Kwon J., Kim K., Ko S. H. (2015). Sci. Rep..

[cit34] Jung J., Kim K. K., Suh Y. D., Hong S., Yeo J., Ko S. H. (2020). Nanoscale Horiz..

[cit35] Kim M., Kim D.-J., Ha D., Kim T. (2016). Nanoscale.

[cit36] Won S., Jung H.-J., Kim D., Lee S.-H., Kim H.-D., Kim K.-S., Lee S.-M., Seo M., Kim D.-S., Lee H.-J. (2020). et al.. Carbon.

[cit37] Dogru-Yuksel I. B., Han M., Pirnat G., Magden E. S., Senses E., Humar M., Nizamoglu S. (2020). APL Photonics.

[cit38] Ghomian T., Hihath J. (2022). IEEE Trans. Biomed. Eng..

