# Supplementary material for: Origami nanogap electrodes for reversible nanoparticle trapping
Source: Nanoscale. 2024 Mar 22;16(17):8514–20. doi: 10.1039/d4nr00190g (PMC11064776; doi:10.1039/d4nr00190g)
Supplement: NR-016-D4NR00190G-s001 [file NR-016-D4NR00190G-s001.pdf]

Electronic Supplementary Material (ESI) for Nanoscale.
This journal is © The Royal Society of Chemistry 2024

Supplementary information for: Origami Nano-gap Electrodes for Reversible Nanoparticle Trapping

ITIR BAKIS DOGRU-YUKSEL, ALLARD P. MOSK, SANLI FAEZ

We determined the crack intervals through the analysis of scanning electron microscopy (SEM) images, considering 40 crack intervals. The size distribution of cracks intervals is shown in Figure S1-a. The average size is $w_r = 15.5 \pm 4.4$ μm. Knowing the average size of the interval regions allows us to estimate the crack gap size for each radius of curvature of the bent film using $d_g = t_f w_r / R$, with R the bending radius and t_f the PET- substrate film thickness. The estimated value is 100 ± 30 nm while the trapping crack gap is measured 258 ± 95 nm according to Figure S1-b. We observed that crack gap varies between 75 nm to 475 nm.

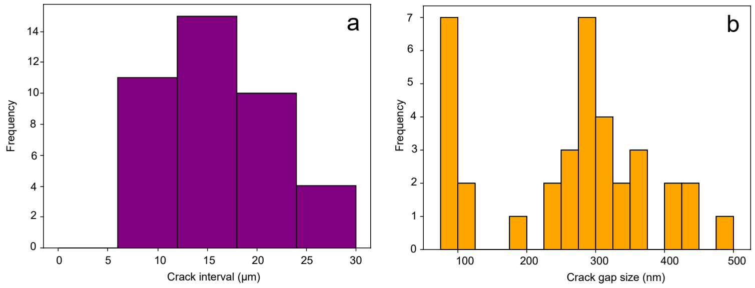

Fig. S1. Length distribution of (a) crack intervals and (b) single trapping crack gap.

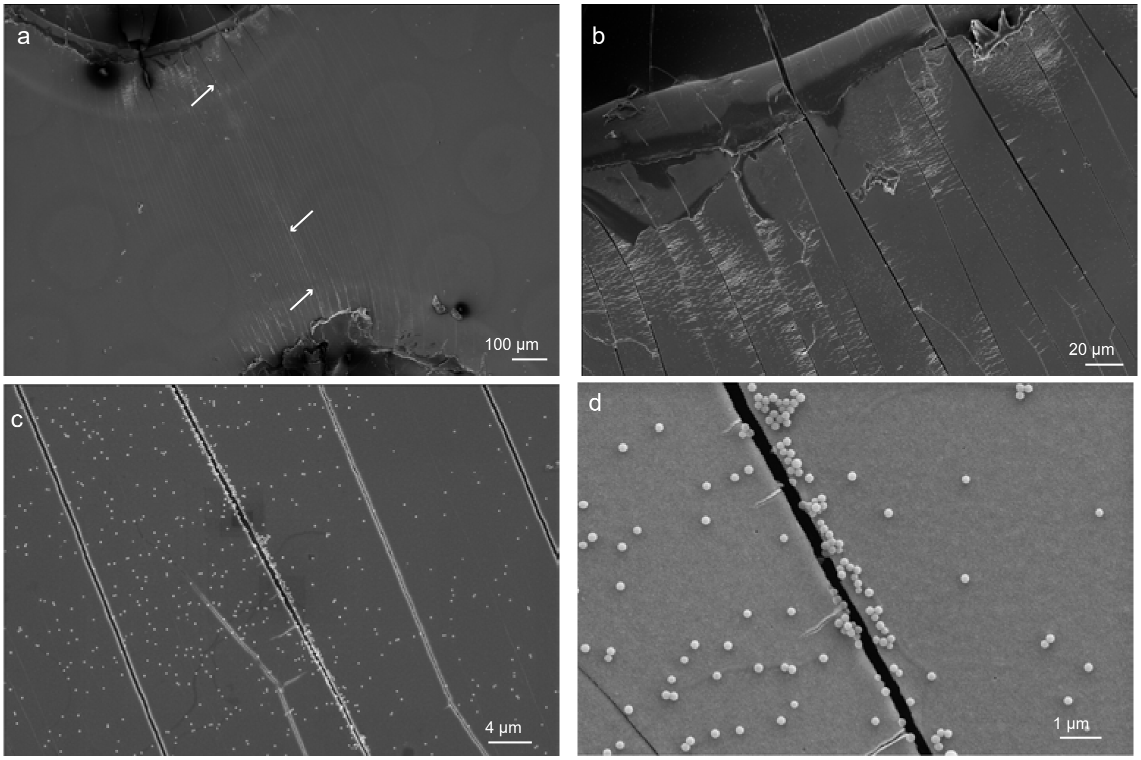

Fig. S2. SEM images of a complete crack that traps the particles. (a) All the origami nanocracks on electrode where the trapping crack is marked with white arrows and (b) its zoomed version at the initiation point. (c) Same crack image taken from a mid-region and (d) its zoomed version.

SEM images in Figure S2 were captured from the samples used in the trapping experiments. In Figure S2-a, the entirety of the cracks is prominently visible, providing a comprehensive view of the structural details. Despite their uniform parallel arrangement, the pivotal aspect lies in their ability to effectively trap particles when the crack separates the conductive layer. The white arrows in the images highlight the nanocrack that effectively trapped nanoparticles, evidenced by the presence of particles along their paths. Despite the existence of numerous nanocracks, as depicted in Figure S2-b, it is crucial to note that they do not share a common initiation point. Notably, those lacking trapping properties are distinguishable by their inability to completely separate the conductive layer on the two sides, consequently failing to create an electric field for trapping because of the electric shortcut. Figure S2-c provides a depiction of the same nanocrack from a mid-region, revealing an array of particles along both sides and the top facet of the crack. This perspective offers a comprehensive view of the spatial distribution of particles within the crack. Further magnification of this region is presented in Figure S2-d, providing a detailed zoomed-in portrayal.

Alternatively, in instances where there are multiple complete cracks, simultaneous particle trapping is observable within each of them (Figure S3). The presence of more than one complete and uniformly patterned nanocrack allows for the creation of multiple particle arrays, further showcasing the versatility and effectiveness of our origami-inspired electrode design.

Detailed insights into reversible particle trapping, even at frequencies as low as 10 kHz, are presented in Figure S4. Prior to the application of an electric field (Figure S4-a), the particles exhibit random movement. However, upon the application of a 5V p-p field at 10 kHz, a notable transformation occurs as the particles align along the nanocrack, as depicted in Figure S4-b. Significantly, when the electric field is discontinued, the particles are swiftly released, as visually confirmed in Figure S4-c. This release phenomenon is further quantified through intensity integration along the entire crack, as illustrated in Figure S4-d. The green hashed regions correspond to the period when the field is active, while the black data trace represents the intensity changes. Notably, the intensity increases rapidly during the field activation and decreases swiftly

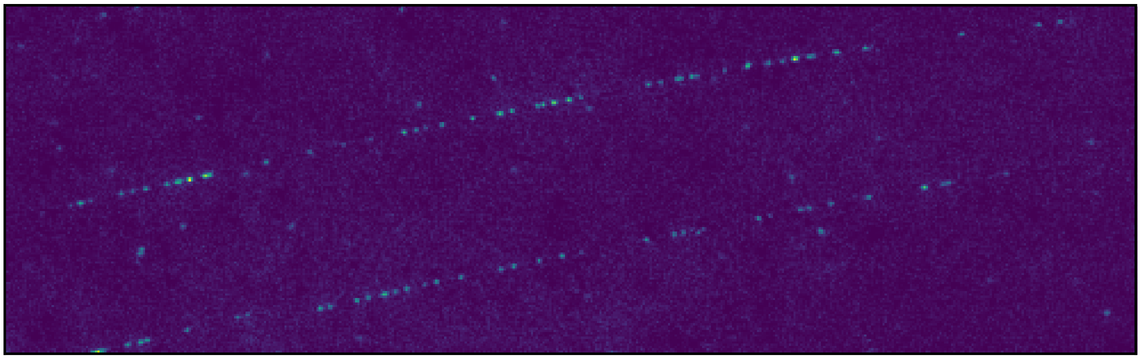

Fig. S3. Two parallel cracks that works simultaneously for particle trapping.

upon field termination, affirming the dynamic reversibility of our particle trapping mechanism.

In addition, an in-depth examination of particle behavior across various frequencies (100 kHz, 10 kHz, 1 kHz, 100 Hz, 10 Hz, and 1 Hz) was undertaken while maintaining a consistent applied potential of 5V during 5 seconds. The application time slot is visually marked by the green indicator that slightly change (Figure S5).

The integrated intensity along the cracks was calculated, with the black data illustrating the nuanced changes in particle numbers exclusively along the cracks. Grouping the particle movement based on frequency, we observed reversible trapping from 100 kHz to 10 kHz (Figure S5-a and b). Notably, at these frequencies, the intensity increased during field application, indicating particle positioning along the cracks, and decreased upon field termination, signaling their release. The critical threshold below which trapping ceases is approximately at 3 kHz.

At 1 kHz and 100 Hz, particles exhibited rapid movement away from the cracks, appearing as if they were actively pushed away (Figure S5-c and d). Although this displacement occurred too swiftly for detailed camera detection, a distinctive and sharp intensity decrease was observed within the crack region at the initiation of the field application. This phenomenon suggests a dynamic response to the field, where particles are pushed away from the trapping sites.

At 10 Hz and 1 Hz, the camera successfully captured particle movements resembling a pendulum, oscillating on both sides of the cracks in accordance with the respective frequencies (see Figure S5-e and f). This observation is further highlighted by the presence of sharp peaks occurring at regular intervals, corresponding to each period of the frequency. This comprehensive frequency analysis offers valuable insights into the nuanced dynamics of particle manipulation within our origami-inspired electrode system.

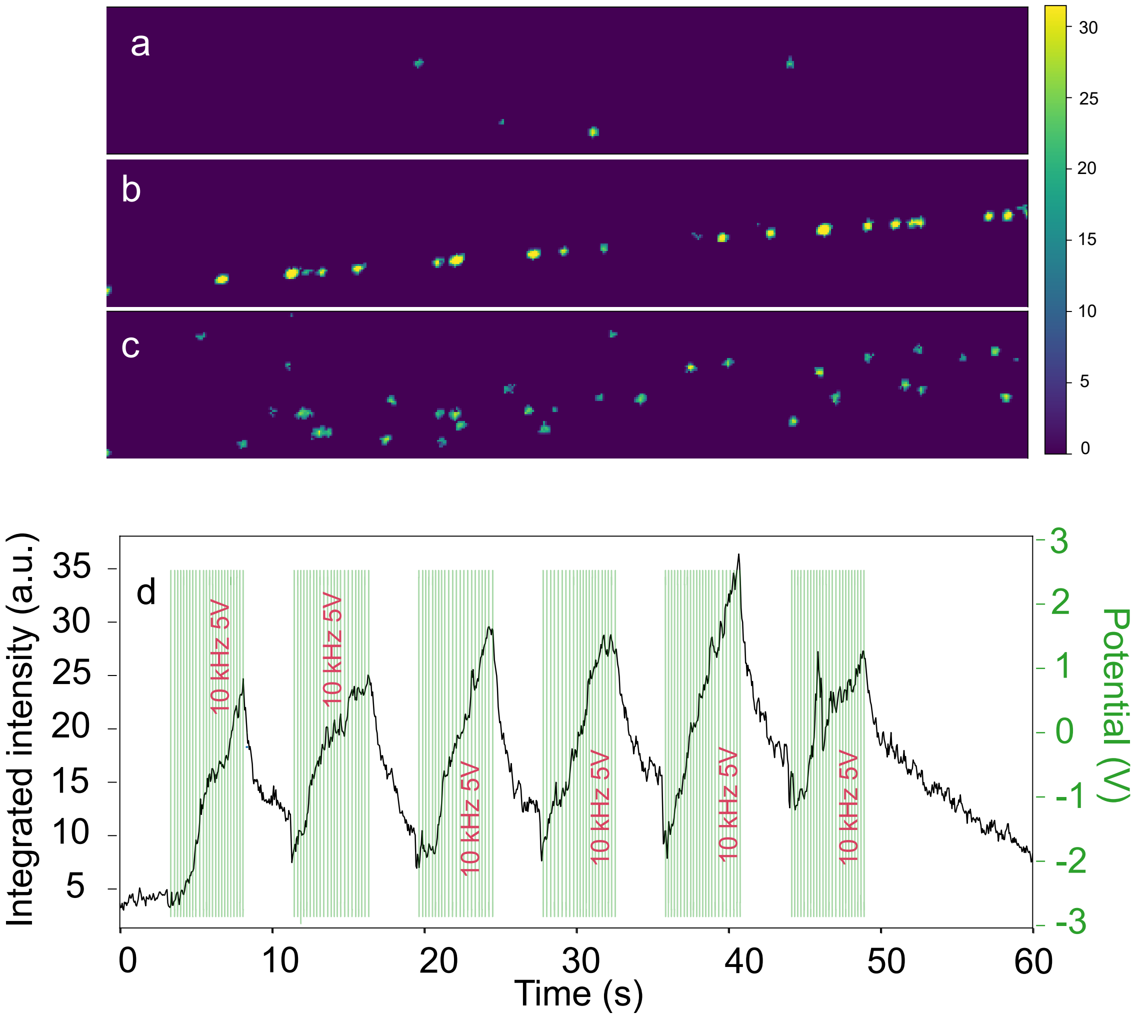

Fig. S4. (a) Before, (b) during and (c) after electric potential application to the origami electrode at 5V at 10 kHz. (d) Integrated intensity change over time in terms of potential field application and cut.

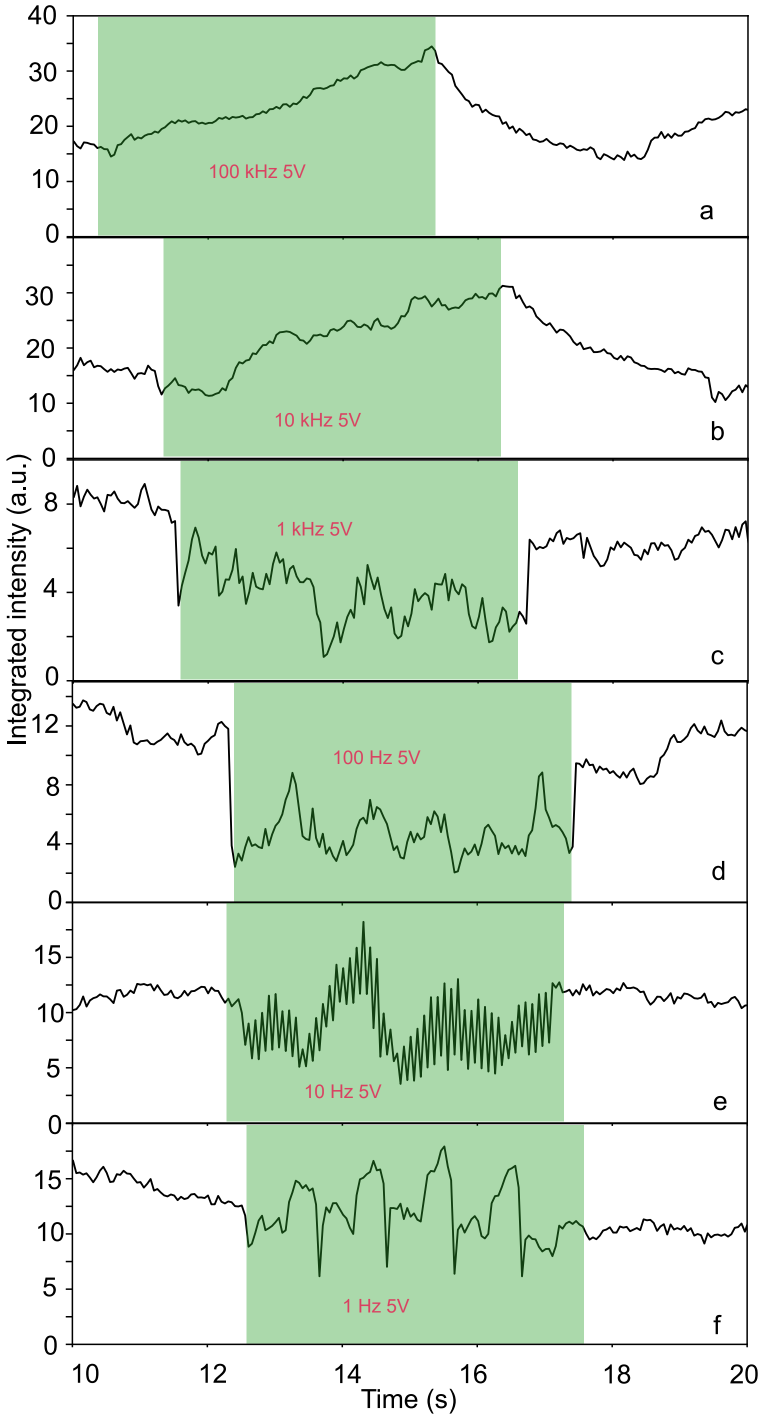

Fig. S5. Integrated intensity change over time during electric potential application at 5V for various frequencies: (a) 100 kHz, (b) 10 kHz, (c) 1 kHz, (d) 100 Hz, (e) 10 Hz, and (f) 1 Hz, with the field application times highlighted in the green region.
